# Pattern and outcomes of medical malpractice cases in Ghana: a systematic content analysis

**DOI:** 10.4314/gmj.v56i4.11

**Published:** 2022-12

**Authors:** Jonathan Bayuo, Adwoa O Koduah

**Affiliations:** 1 School of Nursing, The Hong Kong Polytechnic University, Kowloon, Hong Kong SAR; 2 Department of Nursing, Faculty of Health and Medical Sciences, Presbyterian University College, Ghana

**Keywords:** healthcare, malpractice, legal precedents, negligence

## Abstract

**Objectives:**

Medical malpractice complaints are apparently on the rise in Ghana. Though it has been suggested that medico-legal training programmes should emphasise the kinds of legal problems that healthcare staff encounter most frequently in practice, no comprehensive study currently analyses the pattern and outcomes of existing case laws.

**Design:**

Systematic content analysis

**Data sources:**

Medical malpractice case laws sourced from Ghanaian legal repositories, media platforms and other grey literature sources.

**Results:**

Nine case laws were retained. Most of the cases (n=7) involved negligence. Emerging patterns of cases are complex, including patients' access to their medical records, practising without a license/ out of scope, refusal to treat, and the development of complications following surgical interventions. Obstetrics & Gynaecology, Surgery, and Paediatrics were the main clinical specialties involved in the malpractice cases identified.

**Conclusions:**

The pattern of the cases suggests that all medical specialties are potentially at risk, although most of the cases emerged from Obstetrics & Gynaecology, Surgery, and Paediatrics. Medico-legal training for healthcare staff should emphasise the duty of care and adherence to the Ghana Health Service Patient Charter.

**Funding:**

None declared

## Introduction

Healthcare practice has evolved significantly over the past decades, becoming increasingly complex, albeit its goals of saving lives, alleviating suffering, and maintaining patient dignity have remained unchanged.^1^ Irrespective of how healthcare practice evolves, it will continue to be regulated by context-specific legal and ethical norms, which makes it imperative for healthcare staff to be knowledgeable about these frameworks to avoid malpractice complaints;^2^,^3^ as highlighted by the legal maxim: *ignorantia juris neminem excusat* (ignorance of the law is no excuse). Nonetheless, healthcare staff must often make major clinical decisions under strenuous conditions based on reasonable medical probability. The clinical decision-making process can be even more challenging in low-and-middle income settings (LMICs), where there are numerous challenges, such as time pressures and poor healthcare infrastructure.^4^ Patients may therefore be pre-disposed to harm, creating opportunities for malpractice issues.^5^

Medical malpractice occurs when a hospital, doctor, or other healthcare staff, through a negligent act or omission, causes an injury to a patient. Malpractice represents the intersection of medicine and law and can be categorised into two forms: tort or personal injury law which requires proof that the defendant owed a duty of care to the plaintiff and that the defendant breached this duty by failing to adhere to the expected standard of care, and that the breach of duty caused an injury to the plaintiff^6^ and criminal law which is rare and requires egregious actions that violate a country's criminal code.^7^ Medical malpractice complaints (particularly those in the tort category) are an increasing phenomena across the globe.^2^ In the United States, up to 7.4% of healthcare staff are accused of malpractice each year.^8^ Up to 4% of the 108,000 medical practitioners insured by a German insurance company experienced a malpractice complaint in 2009. 2 In the United Kingdom, malpractice issues involving general practitioners have increased more than two-fold from 2007 to 2012.^9^ In South Africa, up to 2,403 malpractice complaints were received between April 2011 and March 2012.^10^ Though data regarding medical malpractice are lacking in other African countries, news across various media platforms suggest that the phenomenon is potentially on the rise. Most of these malpractice complaints are related to the development of complications following treatment, adverse doctor-patient interaction such as informed consent,^2^ misdiagnosis, practicing outside one's scope, and refusal to treat a patient.^10^ Also, the most frequently reported specialties include Obstetrics and Gynaecology, Emergency Medicine, General Surgery, Orthopaedics and Traumatology.^2^

Although malpractice complaints may seek to advance patient empowerment 11–13, the consequences are often far-reaching. Patients often have no clear idea why they have been injured by their medical treatment and may require psychological support.^14^ One study reported that being involved in a medical malpractice case remains one of the most stressful events in a physician's career, which can even lead to revoking of one's professional license.^15^ Healthcare staff experience emotional distress irrespective of the outcome of the malpractice case.^16^ The risk of lawsuit can also lead to fear among practitioners affecting their approach to patient care.^5^ Damage claims against healthcare institutions can also affect their financial reserves which can be burdening considering that facilities, particularly in LMICs face financial constraints.^10^, ^17^ Thus, the intersection of law and medicine remains a real battle.^5^ The risk of lawsuits among healthcare staff may push them to be better educated to avoid such instances altogether.^18^

Ghana, a developing country, is equally faced with an increasing concern regarding patient safety.^5^, ^11^ Patients are generally considered to be vulnerable, that is, they are unable to protect themselves against threats to their integrity due to their diseases or injuries.^5^, ^11^ What is more, healthcare staff may often be unaware of the nuanced nature of patient vulnerabilities with a general lack of the workings of the law as it applies to medical practice. Ghanaian healthcare staff generally have low levels of legal knowledge and those that are delivered as part of their training have been described as insufficient^1^ which reflect global trends.^3^, ^19^, ^20^

Recently, a basic medical law curriculum has been proposed in Ghana focusing on the kinds of legal problems that physicians encounter.^1^ Court case-based education is generally considered effective in promoting patient safety as it focuses on real cases 21, 22 Analysing the real-life court cases of malpractice can help healthcare staff to uncover the causes of harm/ injuries as well as violations of legal obligations and responsibilities.^23^, ^24^ Despite this assertion, there is currently no comprehensive review in the Ghanaian jurisdiction that pools malpractice cases which have been decided upon in the courts. This is a significant gap as without knowing the pattern and outcomes of these malpractice cases, it may be difficult to ascertain the common legal problems encountered by practitioners in Ghana. The study sought to identify the pattern and outcomes of medical malpractice cases in Ghana.

## Methods

### Design

Systematic content analysis^25^ was adopted for this study. The systematic content analysis approach is an established technique in legal studies applied to case laws to identify patterns of judicial opinions.^25^, ^26^ Inspired by the epistemological roots of legal realism, systematic content analysis focuses on the law as it appears in reality and as such, shaped by how judicial, and administrative bodies administer legal rules.^27^

### Case selection

Five Ghanaian legal repositories were searched by both authors independently for case laws decided upon by a Ghanaian court or a national quasi-judicial body: 1) Ghana Law Finder/ Reports (hosted by the Judicial Service of Ghana) 2) Ghana Legal Information Institute 3) Ghana Law Hub 4) LawsGhana and 5) GhanaJustice. Grey literature sources across media platforms in Ghana were electronically searched for malpractice cases/ complaints. The databases of the healthcare professionals' regulatory bodies in Ghana were also searched for court cases/ rulings involving medical malpractice: 1) Medical and Dental Council, Ghana 2) Nursing and Midwifery Council 3) Pharmacy Council, Ghana 4) Allied Health Professions Council and 5) Ghana Psychology Council. The following search terms were employed: “malpractice” OR “negligence” OR “legal” OR “law” AND “healthcare workers” OR “nurses” OR “medical workers” OR “healthcare professionals” OR “healthcare staff” OR “physicians” OR “doctor”.

The eligibility criteria were malpractice cases ruled by the courts or a national quasi-judicial body. Cases that were handled by regulatory bodies were therefore excluded. Cases that are still pending in court were also excluded. No limits were established regarding the year of occurrence or court ruling.

### Selection of sources of evidence

All identified cases were pooled to EndNote X9.2, following which de-duplication was carried out. This was followed by screening the cases to identify aspects related to the study aim.

### Coding

The retained cases were read thoroughly by both authors and required data pooled using a data extraction tool by JB and reviewed for completeness by the second author (table 1). Pooled data included the case title/ number, year of the case, case summary, legal principles employed, specialty involved, any defenses raised, dissenting opinion, and the holding/ court ruling (outcomes) which are congruent with the overall aim of the review (see Table 1). All the studies were coded independently by both authors to identify the pattern of the cases, judicial reasoning, and outcomes. In case of disagreement, the authors discussed the issue to attain consensus.

**Table 1:** Characteristics and summary of included cases

Caselaw	Case summary	Legal principle	Healthcare staff/ specialty	Defenses	Dissenting opinion (if any	Findings	Outcome/ ruling (the holding)
**State V K.** **Nkyi** **[1962]** **GLR 197**	A student nurse mistakenly injected a baby with arsenic instead of mepacrine. The child's condition immediately deteriorated and died within a few hours. A post-mortem examination revealed that the death of the sick child was caused by arsenic poisoning.	Negligence (practicing without a license)	Nursing	None raised	None	The student nurse was practicing without possessing the requisite registration as a nurse or under the supervision of a qualified practitioner, when he administered a drug to the sick child.	The court held the student nurse liable for the charge of manslaughter.
**Asafo V.** **Catholic** **Hospital of** **Apam** **[1973] 1** **GLR 282**	The plaintiff's six-week-old daughter was admitted at the defendant's hospital. On or about 14 January 1970 the child disappeared, and nobody knew her whereabouts.	Negligence; res ipsa loquitur	Paediatrics	The defendants invited the court to consider the security situation at the hospital.	None	The hospital failed to offer a sound explanation for the occurrence. The court reasoned that a child of six weeks old was no different from an inanimate object which was incapable of independent movement but depended on the support of whoever had its custody.	The court held that the doctrine of *res* *ipsa loquitur* could be applied. Also, on the evidence the plaintiff was entitled to damages but to place a monetary value on a human being was against public policy.
**Asantekramo,** **alias Kumah** **v. Attorney-General** **[1975] 1** **GLR 319**	A nineteen-year-old woman who was diagnosed with ruptured ectopic pregnancy underwent an urgent surgical operation at the Komfo Anokye Government Hospital. While the surgery was successful, her right arm became swollen and gangrenous after being transfused an amount of blood by the nursing staff through a vein in that arm. To save her life, her arm was amputated. Two years later, the woman sued the State, seeking damages for negligence on the part of the hospital staff.	Negligence; res ipsa loquitur	Obstetrics & Gynaecology; surgery	The defense raised by the testifying surgeon that the occurrence was a 'mystery' was quashed by the court.	None	The expert evidence showed that the bacteria that caused the gangrene was either transmitted through the blood transfusion needle or a dextrose infusion administered to the woman.	The Court held the State liable for the negligence of the hospital; damages awarded to the plaintiff
**Gyan v.** **Ashanti** **Goldfields** **Corporation** **1 GLR** **466 (1990)**	The plaintiff took his one-year-old son to the defendant company's hospital with a complaint of high body temperature. A senior nurse who believed that the child's presenting history was suggestive of malaria infection administered a chloroquine injection without prior test or consultation with the doctor on duty. As a result of the injection, the child suffered paralysis of his right leg. It was later confirmed that the child rather had polio and the chloroquine injection complicated the condition thereby causing paralysis.	Negligence; Bolam's test; practicing out of scope; res ipsa loquitur	Nursing	The defendant denied liability on the ground that under normal conditions where there was no polio epidemic, as was the case at the material time, the incidence of polio was so low as compared with that of malaria because of the small risk of paralysis from polio. Therefore, there was nothing irregular about the decision of the nurse to administer the chloroquine injection which was the proper remedy for malaria.	None	Both the trial court and the Court of Appeal accepted the defendant's explanation that where there was no polio outbreak, incidence of polio was very low when compared to malaria, and that given the high mortality rate in children suffering from malaria, a medical officer would not normally withhold an injection for the treatment of malaria even though there was a small risk of paralysis if it turned out to be polio. The core of the defendant's argument was that had a doctor been informed, he would likely have administered chloroquine, since malaria was a common cause of admission of infants at the material time.	The trial court held that the plaintiff failed to prove that the paralysis was attributable to any omission or negligent act of the defendants as he failed to lead any evidence to substantiate his allegation that the nurse had failed to follow the medical regulations in place. In the Court of Appeal, however, the nurse was found negligent for playing the role of the doctor. The hospital was also held vicariously liable.
**Darko v** **Korle-bu** **Teaching** **Hospital,** **Suit No.** **AHR 44/06** **[2008]**	A young male reported for treatment at the defendant hospital with a history of pain in his right knee, which on assessment was diagnosed as torn patella ligament. He was requested to sign a consent form to allow a surgical repair of that ligament. Instead of the right knee being operated on, the surgeons operated on the left knee of a patient. The hospital refused to further attend to the patient as a protest over a medical negligence suit the patient had initiated against them	Negligence, refusal to treat	Surgery		None	The court adopted the Bolam principle and found that the hospital had not been negligent when the left knee was rather operated on. It was observed by the court that the patient had signed a broad consent form which empowered the surgical team to take any necessary measures for the purpose of the operation. Accordingly, if there was a medically justifiable indication for the operation of the left knee, the hospital could not be found negligent for treating it. The court also pointed out the failure of the plaintiff's lawyer to advance arguments on the scope of the consent given vis-à-vis the medical complaint reported by the boy. However, the hospital was found in breach of its duty to provide the boy care when it refused to honor his review and physiotherapy appointments during the pendency of the suit as a protest to his legal action.	The court did not find the doctors or the hospital liable for negligence in operating on the left knee instead of the right but did find that the hospital was liable for refusing the claimant further treatment after the legal action had been initiated.
**Elizabeth** **Vaah v Lister** **Hospital** **and** **Fertility** **Centre,** **suit number** **is** **HRCM** **69/10** **[2010]**	A client who was under the care of the defendant hospital sued the hospital, relying on the right to information guaranteed under Article 21(1)(f) of the 1992 Constitution of Ghana (the Constitution), when she sought to recover her medical record to clarify the cause of death of her stillborn baby. The applicant's case is that her fundamental human rights have been violated by the respondent when the latter refused to release her medical records to her.	Violation of right to personal records	Hospital/ physicians	The respondent argued that it was justified in refusing the applicant's request for medical records because by speaking to the press about the circumstances in which she gave birth at the respondent hospital, she had evinced an intention to abuse the records.	None	The court analysed the constitutional provision on freedom of information and noted that the excuse provided by the respondent in denying access to the applicant was not covered by the qualifications contemplated by the Constitution for limiting freedom of information.4	It was held that the plaintiff was entitled to a copy of her medical record from Lister Hospital.
**Somi v** **Tema General** **Hospital,** **(1994–2000)** **CHRAJ** **196**	a 36-year pregnant woman was rushed to hospital with an ante partum haemorrhage. The doctor on night duty had finished earlier than expected at 4.00 a.m. instead of 8.00 a.m. and the morning doctor on day duty did not report until 10.00 a.m. The nurses tried to keep the patient alive, but they could not hear the heartbeat of the unborn child. Neither the mother nor the baby survived the operation.	Negligence: abuse of official time-absence /lateness to duty	Obstetrics & gynaecology; surgery	None	None	CHRAJ found the defendant hospital to have unjustly caused a patient's death in violation of Article 218(a) of the Constitution.	It was held that the failure of a public hospital to ensure that an emergency caesarean section operation was carried out on a patient, thus leading to her death, constituted a violation of her human right to life.
**Kwaku** **Agyire-Tettey &** **Paul** **Kwaku** **Sodokeh v.** **The University** **of** **Ghana & 2** **Others** **[2018]**	The plaintiff's late wife underwent treatment for fertility issues at the University of Ghana Hospital before she got pregnant and utilised ante-natal services at the same hospital where she was booked to undergo a caesarean section. According to the plaintiff, his wife with "her knowledge of customer service in the medical field from her previous job as a Customer Service Lecturer for Doctors and Nurses enquired from both consultants if there were any risks associated with the removal of fibroid during Caesarean delivery and was told it was a normal and regular practice without any risks”. Following the surgery, the plaintiff's wife was discharged around the third post-operative day. Some complications resulted following discharge which led to readmission of the plaintiff's wife but later died at the Korle-bu Teaching Hospital.	Negligence	Obstetrics & gynaecology; surgery	In this case, the Bolam principle was administered and one of the respondents, Dr. Maya in reacting to the Plaintiff's allegation that the deceased should not have been discharged at the time she was discharge , Dr. Maya said "discharging patients who are deemed medically fit on post-operative day three (both obstetric and gynaecological major case) is not peculiar to the maternity ward of the Hospital. Throughout my postgraduate training and beyond, and in all the facilities I have worked, patients are discharged on post-operative day three if they are deemed medically fit”. Other physicians testified for the Defendant as the case in Bolam principle.	None	The court found that "based on all of the evidence that on the balance of probabilities there is no credible evidence that the Defendants' servants were negligent when they treated the deceased as a patient at the University of Ghana Hospital. It is clear that the deceased death cannot be attributed to the doctors who treated her because they fell short of the standard required of them. There is no cogent evidence that the 1st Plaintiff's wife death was due to the negligent actions and/or in actions of the Defendants' servants. In arriving at the above conclusion, I reject the sole evidence of the Plaintiffs proffered by the 1st Plaintiff as bald allegations which were not backed by any acceptable cogent evidence”	The Court, in their decision, ruled out any act of negligence on the part of the Physicians. The defendants were however entitled to some costs though not 50,000 as requested, a nominal amount of 7000 was approved by the judge.
**Jehu Appiah v** **Nyaho** **Healthcare** **Limited**	The plaintiff accused the facility of allegedly damaging her fallopian tube, which nearly led to her death. According to the case, the plaintiff, upon conception utilised antenatal care services at the respondent hospital. But at a point, she claimed she had to undergo a life-saving surgery at a different health facility due to the "actions and inactions" of the Nyaho hospital. After the life-saving surgery, she made a formal complaint to Nyaho Healthcare Limited, after which she was promised investigations into the matter and the results communicated to her. The plaintiff noted that all efforts to compel the respondent hospital to release her medical documents (including scans, tests, diagnosis, and treatment) proved futile.	Handling patient medical records	Obstetrics & Gynaecology	The respondent hospital prayed the court to refuse the application on the following grounds: firstly, the application was not supported by law; secondly, the plaintiff's application did not identify or disclose any other information, not within her knowledge.	None	The court found that the healthcare service provider had not in its defence denied possession and custody of the documents, as such, must release the information.	The court held that the complete medical records be released to the patient. An award of 2000 Ghana Cedis was awarded to the patient.

### Synthesis

The emerging codes from each study were reviewed iteratively. Constant comparison was employed to examine these codes across studies to identify if similarities existed. The patterns, associations, and outcomes across the case laws were noted which formed the basis of undertaking a narrative synthesis.

### Case law characteristics

Nine cases were identified from the extensive search. 28–36 The earliest case was recorded in 1962.^35^ Majority of the cases (n=7) involved negligence 28–30, 32–35 and two cases concerned access to medical records.^31^, ^36^

## Results

Table 1 shows a summary of the cases included in the study.

## Discussion

The analysis sought to identify the pattern and outcomes of medical malpractice case laws in Ghana. Emerging patterns of malpractice cases include patient's access to medical records, practicing without license, practicing out of scope, refusal to treat, and development of complications following surgical interventions. Obstetrics & Gynaecology, Surgery, and Paediatrics were the main clinical specialties involved in the malpractice cases identified which is congruent with a previous study.^2^ Though the cases are limited, it may be suggested that medico-legal training for healthcare staff should emphasise duty of care, practicing within one's scope of clinical practice with the requisite skills expected of a professional at a comparable level, maintaining up to date professional registration with respective regulatory bodies, effective supervision of trainees/ newly qualified staff, effective delegation in patient care, handling patient information/ medical records in line with prevailing standards, and adherence to the Ghana Health Service (GHS) Patient Charter.

In the instance of the State V K. Nkyi [1962] GLR 197, negligence was established based on voluntary assumption of treatment without the requisite skills, qualification or supervision by a qualified practitioner.^35^ Although in a different context, the ruling appears similar to the case of Gyan v. Ashanti Goldfields Corporation 1 GLR 466 (1990) which involved a senior nurse who was found negligent in “attempting to play the role of an experienced doctor on his own”.^34^ In fact, performing a role/ clinical duty outside one's scope, or without the requisite qualification/ certification is a risk not worth taking. Previous case laws in other jurisdictions involving healthcare trainees or inexperienced staff have held that they are judged by the same standards as their experienced colleagues. For instance, In Wilsher v Essex Area Health Authority [1988] 1 All ER 871, the Court of Appeal rejected the claim that an inexperienced junior physician owed a lower duty of care. This legal precedent was subsequently affirmed in FB v Princess Alexandra Hospital NHS Trust [2017] EWCA Civ 334. These rulngs emphasise the need to practice within one's scope of professional training. Though the case involving the State V K. Nkyi happened outside the hospital, there is a potential that student nurses may face a similar challenge during clinical placement particularly in the Ghanaian setting where placement support systems are generally lacking, and student nurses may be left on their own with limited supervision.^37^ These precedents should encourage healthcare facilities to implement effective workplace support programmes for trainees and newly qualified staff. There should also be proper supervision of trainees and newly qualified staff as well as delegation within one's scope of professional practice. The ruling in Gyan v. Ashanti Goldfields Corporation 1 GLR 466 (1990) particularly raises an interest regarding areas in Ghana that may not have access to some healthcare professionals such as physicians, requiring nurses to “play the role of a doctor”.

In Asantekramo alias Kumah v. Attorney-General [1975] 1 GLR 319, the state was held liable for the negligence of the hospital with a subsequent award of damages to the plaintiff. This outcome has been criticised recently by Plange-Rhule noting that the judge may have made certain incorrect interpretations of the medical facts presented as well as failed to appreciate the pathology behind the plaintiff's injury.^38^ This may be related to the fact that normal flora of bacteria also exists within the human body which may be potential aetiologies of the gangrene particularly if the patient had an altered state of immunity. Further to the issue raised is the exclusion of nurses from testifying in this case. This assertion has been re-echoed in a current paper in which the authors highlight it may be related to how nurses were viewed at the time of the case when the Ghanaian nurse relied significantly on the instruction of physicians.^39^ This situation may have evolved over the years as more nurses undergo advanced training, and develop independent practice.

The cases of Vaah v Lister Hospital and Fertility Centre and Appiah v Nyaho Healthcare Limited involve access to medical records. With the ruling in Elizabeth Vaah v Lister Hospital and Fertility Centre, a precedent was set which was also followed in Appiah v Nyaho Healthcare Limited permitting access to their medical records and in the latter case, a nominal amount was also awarded to the plaintiff. These rulings highlight that the patient's right to medical records is protected by the constitution. In both cases, the plaintiffs had to seek legal intervention to have their medical records released to them. The cases reveal a significant gap regarding the lack of a specific framework for accessing health records and a general lack of awareness of healthcare practice in Ghana. Though the GHS Patient Charter established in 2010 may have been consulted in this instance,^13^ it is worth mentioning that the charter only stipulates that “the patient is entitled to full information on his/her condition and management and the possible risks involved except in emergency situations when the patient is unable to make a decision and the need for treatment is urgent” which is rather vague and does not necessarily imply that the “patient” has a right to obtain their medical records after being discharged. Besides, pregnancy is not often considered a ‘disease’. Although the legal precedent is based on the ruling in *Vaal*, a recent study has argued that there is no substantive right of access to medical records and the ruling may have been skewed in favor of the patient.^40^ Yet from the patient empowerment perspective, it may be suggested that such ruling may affirm a joint ownership of medical records between healthcare institutions and patients and as such, patients can access their records when necessary. This confusion may continue till clear frameworks and specific legislations on privacy are enacted in Ghana.

The focus on only case laws decided on by the law courts may be a limitation and future studies may compare the rulings in these cases with those ruled by the respective regulatory bodies in Ghana.

## Conclusion

Medical malpractice cases are emerging in Ghana. The patterns of malpractice cases include access to their medical records, practicing without license/ out of scope, refusal to treat, and development of complications following surgical interventions. Medico-legal training for healthcare staff should particularly emphasise duty of care and adherence to the GHS Patient Charter.
